# Supplementary material for: Integrative metagenomics and structural bioinformatics identify explainable gut microbial variants associated with Crohn’s disease
Source: PLoS One. 2026 Jul 10;21(7):e0340748. doi: 10.1371/journal.pone.0340748 (PMC13354076; doi:10.1371/journal.pone.0340748)
Supplement: S1 Fig — The Venn diagram illustrates the total number of significantly associated species with CD and UC, along with the number of species uniquely and commonly associated with each phenotype. (PDF) [file pone.0340748.s001.pdf]

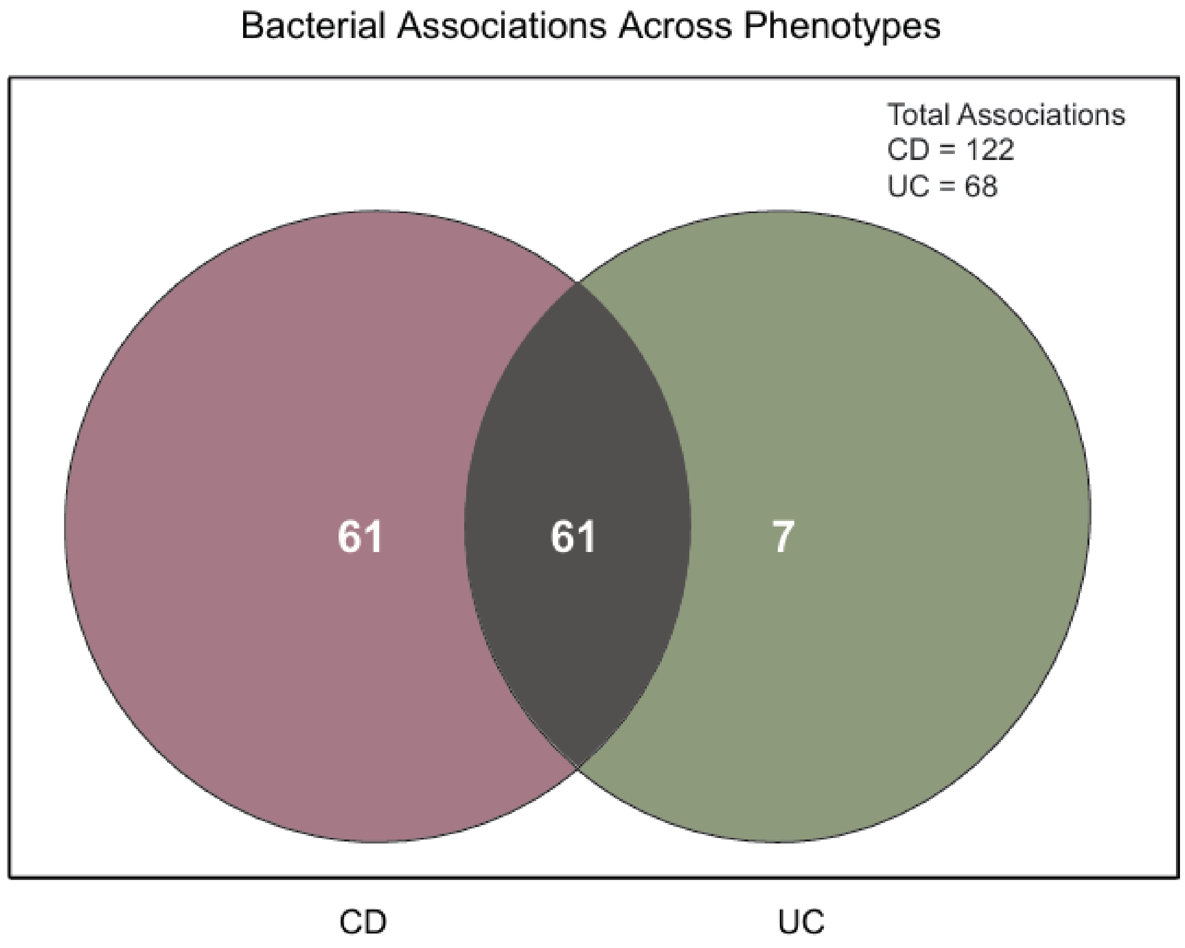

S1 Fig. Bacterial association across phenotypes. The Venn diagram illustrates the total number of significantly associated species with CD and UC, along with the number of species uniquely and commonly associated with each phenotype.
