# Supplementary material for: Integrative metagenomics and structural bioinformatics identify explainable gut microbial variants associated with Crohn’s disease
Source: PLoS One. 2026 Jul 10;21(7):e0340748. doi: 10.1371/journal.pone.0340748 (PMC13354076; doi:10.1371/journal.pone.0340748)
Supplement: S2 Fig — The overall comparative variation number in highly associated bacterial species is shown. SNP variations are high in number in all the species, accordingly with respect to the other variations. (PDF) [file pone.0340748.s002.pdf]

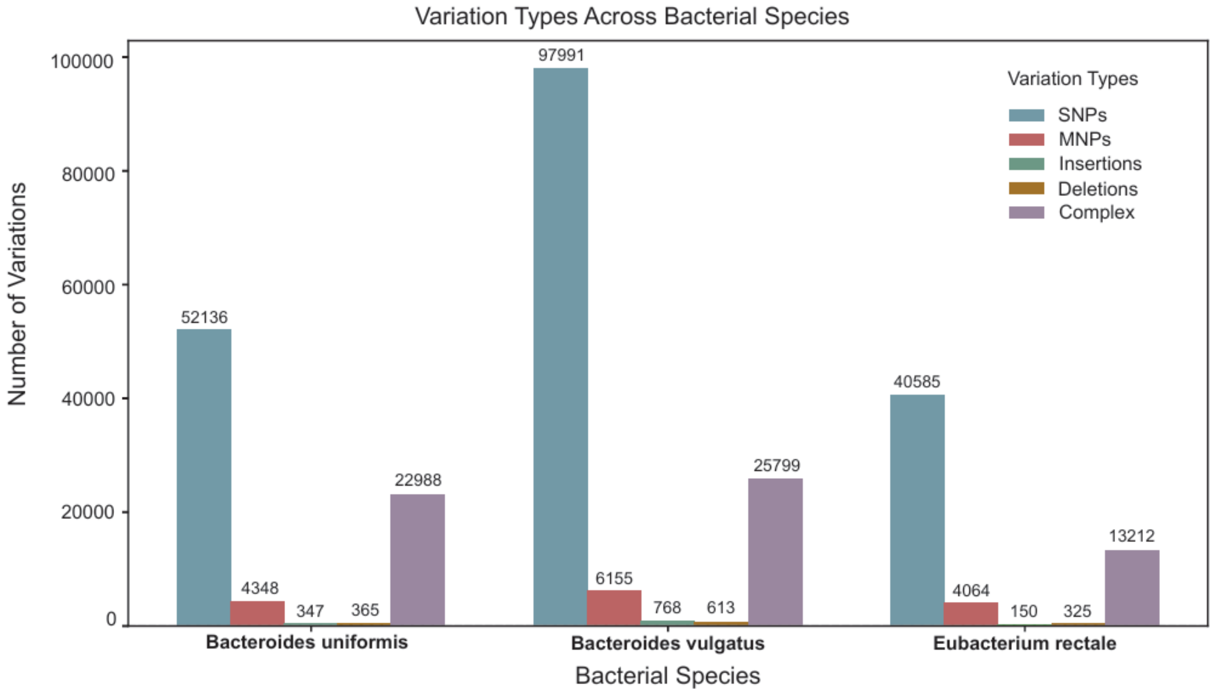

S2 Fig. Variant counts summary. The overall comparative variation number in highly associated bacterial species is shown. SNP variations are high in number in all the species, accordingly with respect to the other variations.
