# Supplementary material for: Integrative metagenomics and structural bioinformatics identify explainable gut microbial variants associated with Crohn’s disease
Source: PLoS One. 2026 Jul 10;21(7):e0340748. doi: 10.1371/journal.pone.0340748 (PMC13354076; doi:10.1371/journal.pone.0340748)
Supplement: S3 Fig — The bacterial species exhibit a variable number of reported SNPs across their genomes, affecting many essential genes that encode specific proteins. These SNPs vary in depth, as shown, and have a significant impact on the corresponding proteins. Notably, a higher number of affected proteins at both lower and higher depths is predominantly observed in CD samples compared to UC samples. (PDF) [file pone.0340748.s003.pdf]

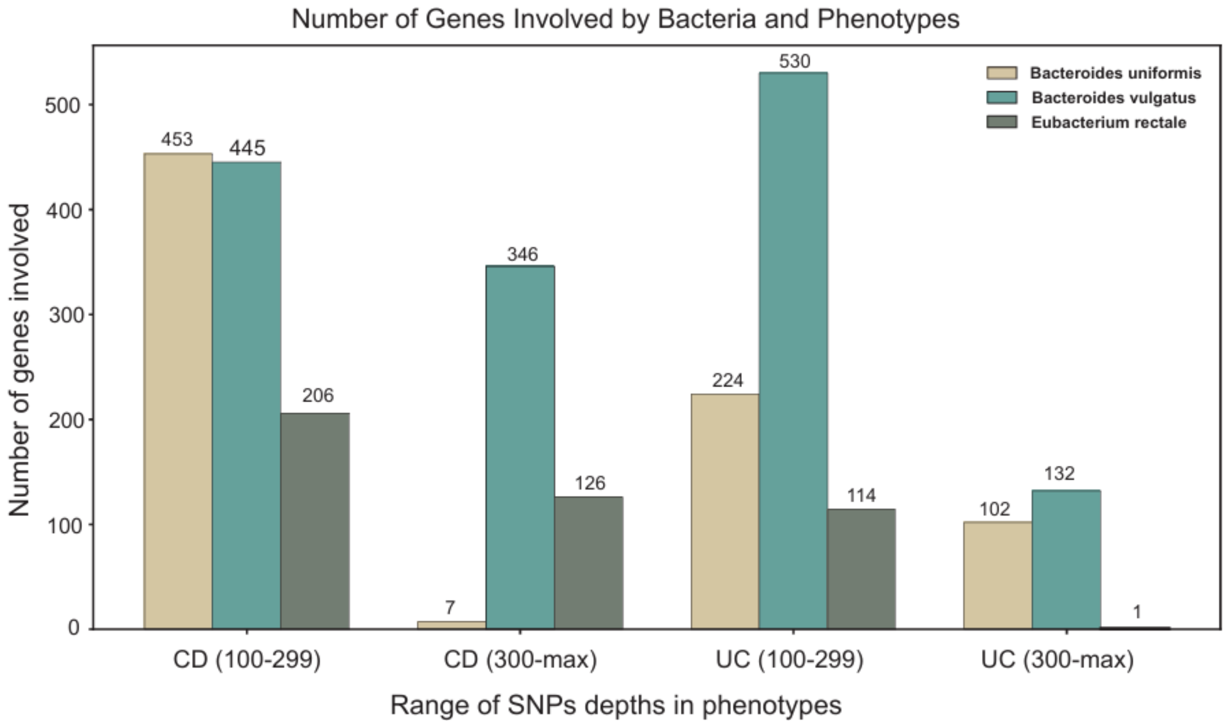

S3 Fig. Catalogue of microbial genes with reported SNPs. The bacterial species exhibit a variable number of reported SNPs across their genomes, affecting many essential genes that encode specific proteins. These SNPs vary in depth, as shown, and have a significant impact on the corresponding proteins. Notably, a higher number of affected proteins at both lower and higher depths is predominantly observed in CD samples compared to UC samples.
