# Supplementary material for: Integrative metagenomics and structural bioinformatics identify explainable gut microbial variants associated with Crohn’s disease
Source: PLoS One. 2026 Jul 10;21(7):e0340748. doi: 10.1371/journal.pone.0340748 (PMC13354076; doi:10.1371/journal.pone.0340748)
Supplement: S4 Fig — The network showed the close interactions of SusD with other proteins (nodes). The thickness of the interacting lines indicated the strength of the association between proteins and SusD. Furthermore, the green nodes, representing the neighboring proteins (see text), show close interactions with SusD, clarifying the intricate role of all the proteins in starch metabolism. Yellow nodes represent hypothetical proteins that might have some contributing associations with SusD accordingly. (PDF) [file pone.0340748.s004.pdf]

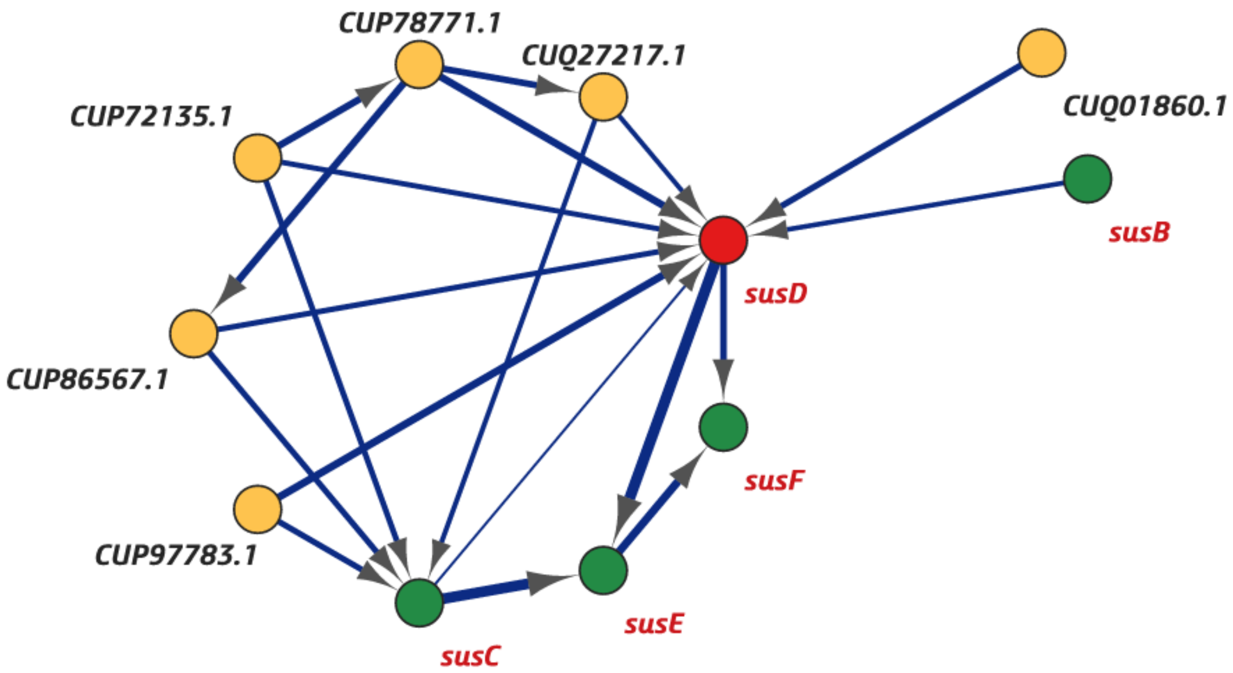

S4 Fig. Network analysis of starch utilization system proteins. The network showed the close interactions of SusD with other proteins (nodes). The thickness of the interacting lines indicated the strength of the association between proteins and SusD. Furthermore, the green nodes, representing the neighboring proteins (see text), show close interactions with SusD, clarifying the intricate role of all the proteins in starch metabolism. Yellow nodes represent hypothetical proteins that might have some contributing associations with SusD accordingly.
