# Supplementary material for: Integrative metagenomics and structural bioinformatics identify explainable gut microbial variants associated with Crohn’s disease
Source: PLoS One. 2026 Jul 10;21(7):e0340748. doi: 10.1371/journal.pone.0340748 (PMC13354076; doi:10.1371/journal.pone.0340748)
Supplement: S5 Fig — The alignment of wild and mutant SusD with the template sequence (3CK7) is shown in (A) and (B). The residues identical to the template residues are represented in black color, further demonstrated by dark green bands. Residues showing similarity to the template sequence are colored grey, while non-matching residues are in white. Gaps are shown to dotted lines. Furthermore, the site where the mutation occurs and its neighboring residues are enclosed in red box, and the alignment results show that most of the residues show no similarity with the template sequence. Holistically, there are multiple residues in the query sequences that show similarity to the template sequence. (PDF) [file pone.0340748.s005.pdf]

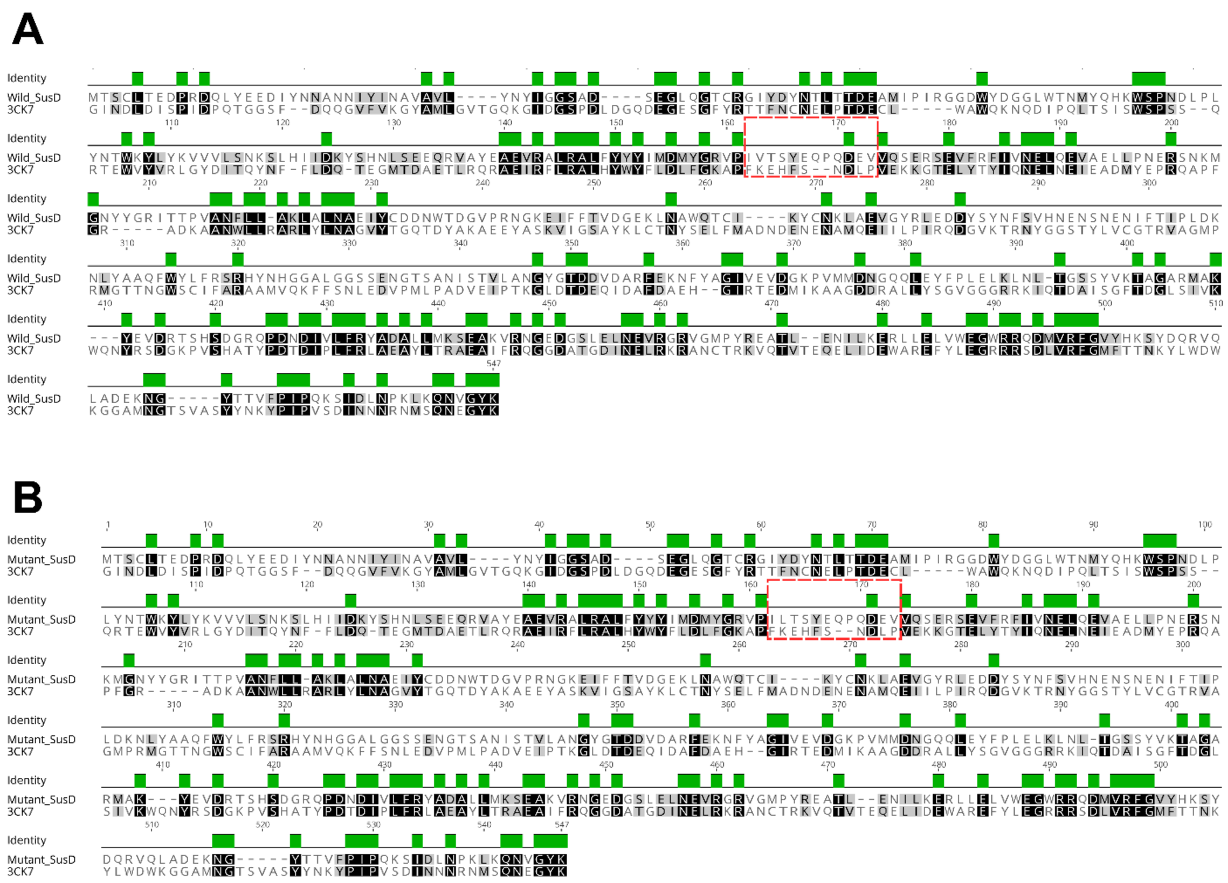

S5 Fig. Sequence alignment of wild-type and mutant SusD with template sequence. The alignment of wild and mutant SusD with the template sequence (3CK7) is shown in (A) and (B). The residues identical to the template residues are represented in black color, further demonstrated by dark green bands. Residues showing similarity to the template sequence are colored grey, while non-matching residues are in white. Gaps are shown to dotted lines. Furthermore, the site where the mutation occurs and its neighboring residues are enclosed in red box, and the alignment results show that most of the residues show no similarity with the template sequence. Holistically, there are multiple residues in the query sequences that show similarity to the template sequence.
