# Supplementary material for: Integrative metagenomics and structural bioinformatics identify explainable gut microbial variants associated with Crohn’s disease
Source: PLoS One. 2026 Jul 10;21(7):e0340748. doi: 10.1371/journal.pone.0340748 (PMC13354076; doi:10.1371/journal.pone.0340748)
Supplement: S6 Fig — (A) illustrate the comparative RMSD of wild and mutant SusD along a 200 ns simulation run. Both proteins initially deviate from their initial conformation at the beginning of the simulation and stabilize (minimal conformational changes) after the 100th time point of simulation. (B) Comparative conformational fluctuations of each residue of wild and mutant SusD are shown along the simulation run, where multiple residues of mutant SusD showed notable conformational changes compared to wild SusD. (PDF) [file pone.0340748.s006.pdf]

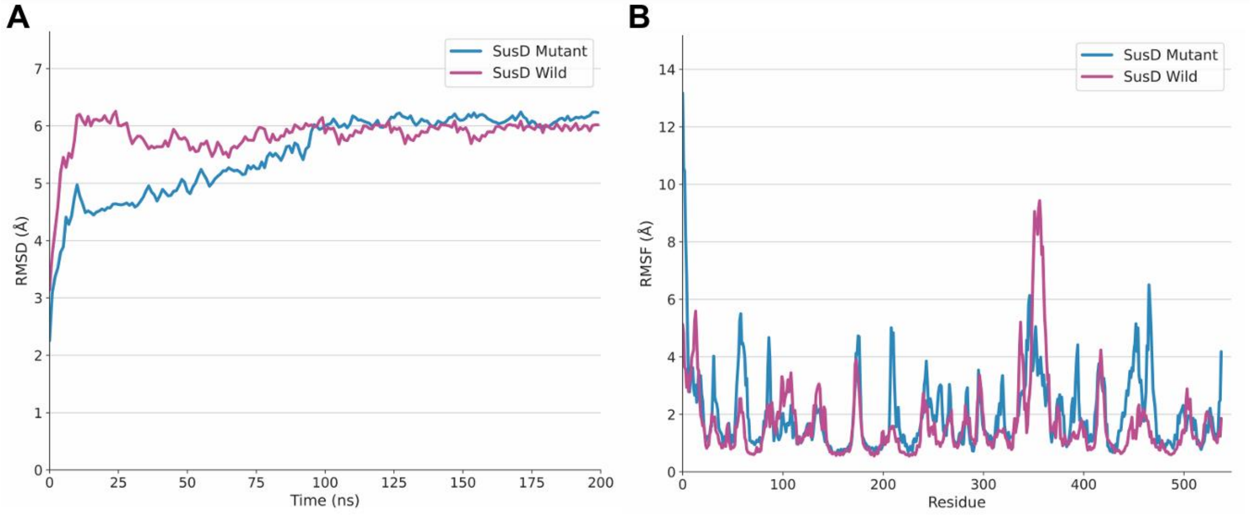

S6 Fig. Simulation of wild and mutant SusD. (A) illustrate the comparative RMSD of wild and mutant SusD along a 200 ns simulation run. Both proteins initially deviate from their initial conformation at the beginning of the simulation and stabilize (minimal conformational changes) after the 100th time point of simulation. (B) Comparative conformational fluctuations of each residue of wild and mutant SusD are shown along the simulation run, where multiple residues of mutant SusD showed notable conformational changes compared to wild SusD.
