# Supplementary material for: Integrative metagenomics and structural bioinformatics identify explainable gut microbial variants associated with Crohn’s disease
Source: PLoS One. 2026 Jul 10;21(7):e0340748. doi: 10.1371/journal.pone.0340748 (PMC13354076; doi:10.1371/journal.pone.0340748)
Supplement: S7 Fig — A cyclic chain of uniformly distributed hydrogen, oxygen and hydrogen atoms. (PDF) [file pone.0340748.s007.pdf]

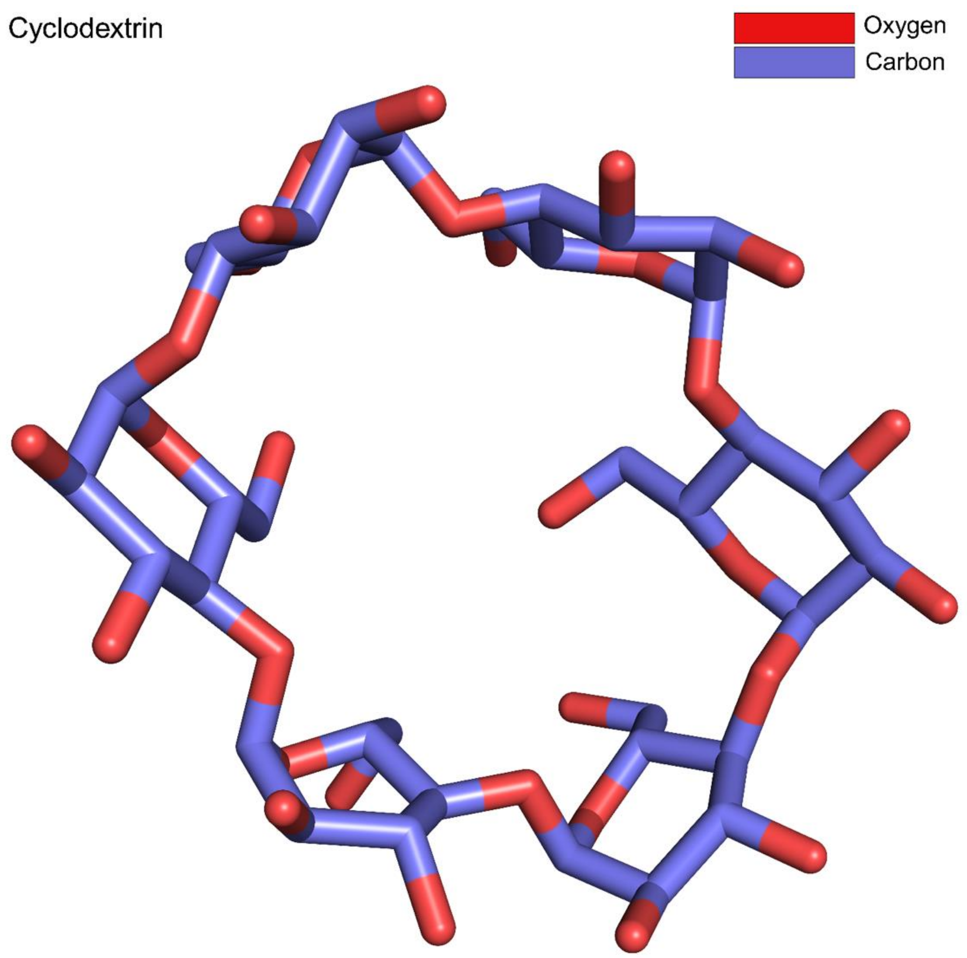

S7 Fig. Structure of cyclodextrin. A cyclic chain of uniformly distributed hydrogen, oxygen and hydrogen atoms.
