# Supplementary material for: Integrative metagenomics and structural bioinformatics identify explainable gut microbial variants associated with Crohn’s disease
Source: PLoS One. 2026 Jul 10;21(7):e0340748. doi: 10.1371/journal.pone.0340748 (PMC13354076; doi:10.1371/journal.pone.0340748)
Supplement: S8 Fig — The plot showed the binding scores of cyclodextrin with wild and mutant SusD across 100 conformations. Each docked conformation of cyclodextrin with wild-type SusD showed a strong binding affinity (more negative energy value) as compared to mutant SusD. (PDF) [file pone.0340748.s008.pdf]

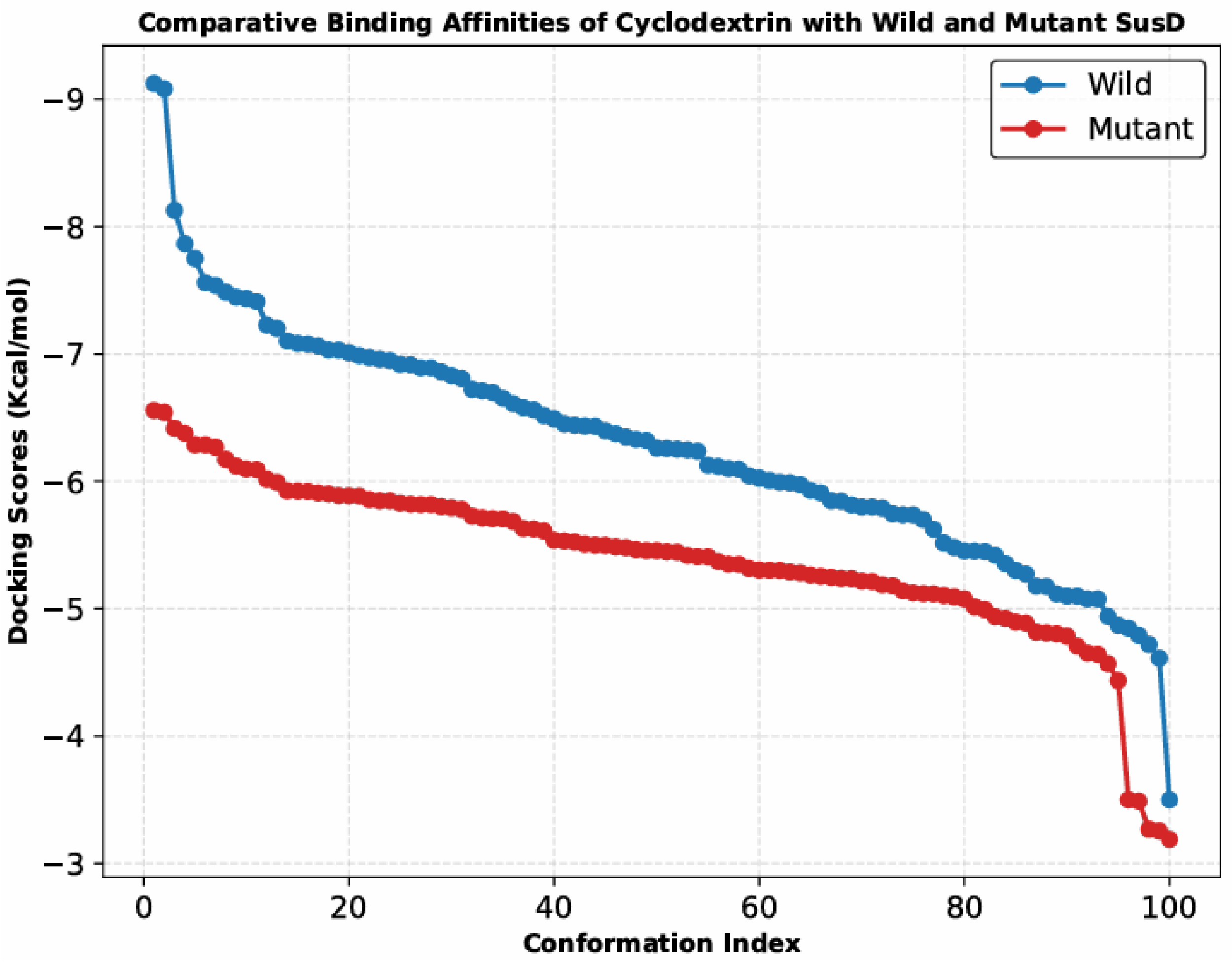

S8 Fig. Comparative docking scores. The plot showed the binding scores of cyclodextrin with wild and mutant SusD across 100 conformations. Each docked conformation of cyclodextrin with wild-type SusD showed a strong binding affinity (more negative energy value) as compared to mutant SusD.
