# Supplementary material for: Integrative metagenomics and structural bioinformatics identify explainable gut microbial variants associated with Crohn’s disease
Source: PLoS One. 2026 Jul 10;21(7):e0340748. doi: 10.1371/journal.pone.0340748 (PMC13354076; doi:10.1371/journal.pone.0340748)
Supplement: S9 Fig — (A) shows the residues of wild SusD involved in non-bonded interactions with cyclodextrin after docking, while (B) presents the interacting residues following molecular dynamics simulation. Similarly, (C) illustrates the residues of mutant SusD contributing to non-bonded interactions with cyclodextrin after initial docking, whereas (D) depicts the corresponding interactions after simulation. (PDF) [file pone.0340748.s009.pdf]

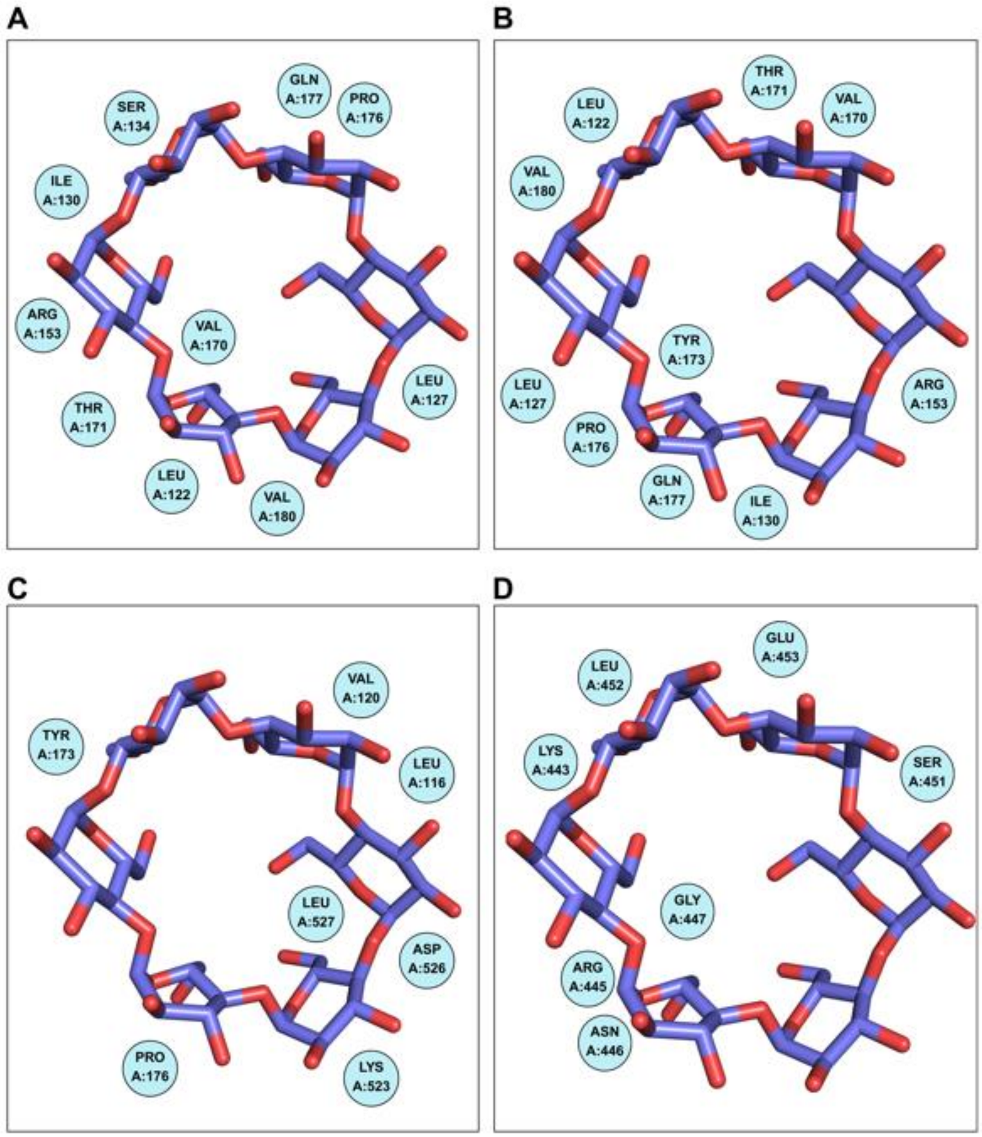

S9 Fig. Non-bonded interaction patterns before and after simulation in wild-type and mutant complexes. (A) shows the residues of wild SusD involved in non-bonded interactions with cyclodextrin after docking, while (B) presents the interacting residues following molecular dynamics simulation. Similarly, (C) illustrates the residues of mutant SusD contributing to non-bonded interactions with cyclodextrin after initial docking, whereas (D) depicts the corresponding interactions after simulation.
