# Supplementary material for: Integrative metagenomics and structural bioinformatics identify explainable gut microbial variants associated with Crohn’s disease
Source: PLoS One. 2026 Jul 10;21(7):e0340748. doi: 10.1371/journal.pone.0340748 (PMC13354076; doi:10.1371/journal.pone.0340748)
Supplement: S10 Fig — Comparative conformational fluctuations of each residue of wild and mutant docked complexes are shown along the simulation run, where multiple residues of the mutant complex showed notable conformational changes compared to the wild-type SusD complex, demonstrating the variable interaction of cyclodextrin with multiple residues of the mutant SusD complex at variable locations. (PDF) [file pone.0340748.s010.pdf]

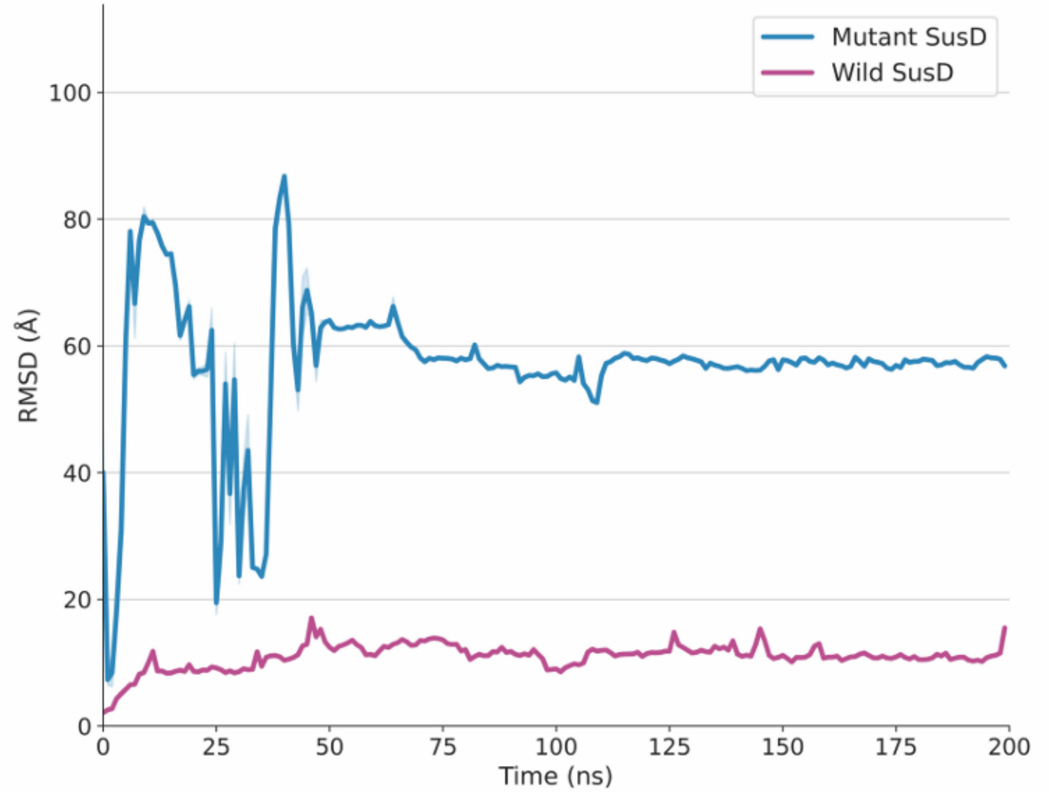

S10 Fig. RMSF of docked complexes. Comparative conformational fluctuations of each residue of wild and mutant docked complexes are shown along the simulation run, where multiple residues of the mutant complex showed notable conformational changes compared to the wild-type SusD complex, demonstrating the variable interaction of cyclodextrin with multiple residues of the mutant SusD complex at variable locations.
