# Supplementary material for: Integrative metagenomics and structural bioinformatics identify explainable gut microbial variants associated with Crohn’s disease
Source: PLoS One. 2026 Jul 10;21(7):e0340748. doi: 10.1371/journal.pone.0340748 (PMC13354076; doi:10.1371/journal.pone.0340748)
Supplement: S11 Fig — (A) Hydrogen bond-forming residues of wild SusD with cyclodextrin over the 200 ns simulation. Residues 172nd to 174th showed consistent interactions throughout, while residues 130th contributed up to 130 ns with minor fluctuation. Additional transient interactions were observed with residues 136th, 176th, and 178th. (B) Mutant SusD residues forming hydrogen bonds with cyclodextrin. Residues 448th and 453rd maintained notable interactions throughout, while residue 174th interacted stably up to 20 ns. Residues 444th and 450th to 452nd exhibited intermittent bonding. (PDF) [file pone.0340748.s011.pdf]

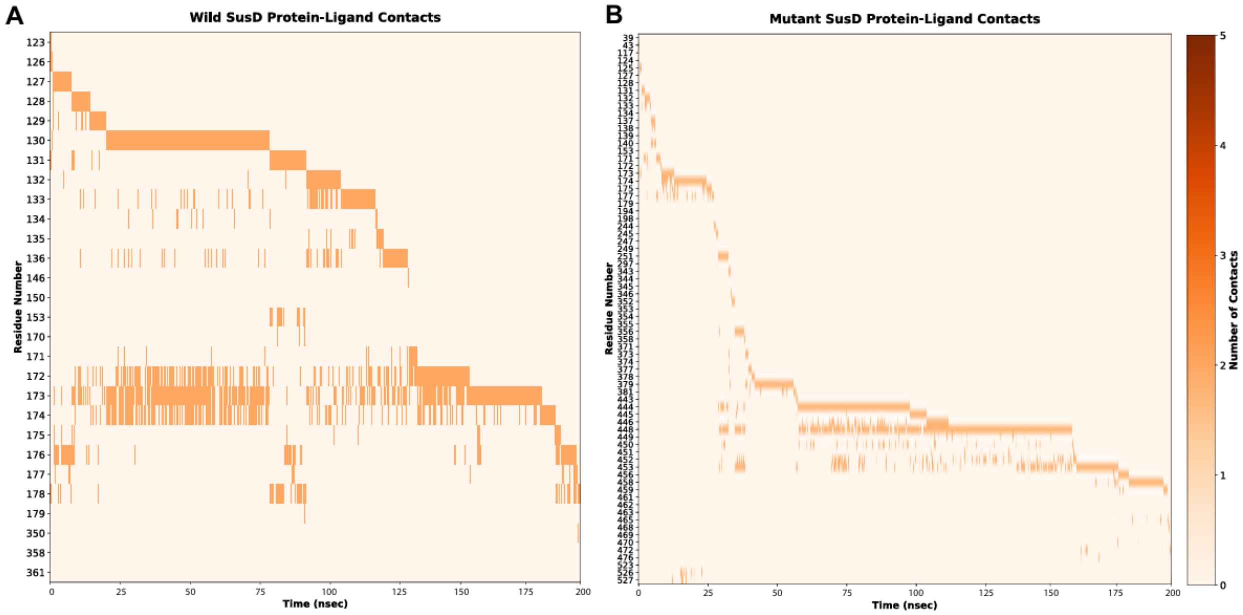

S11 Fig. Hydrogen bonding patterns of wild and mutant SusD complex. (A) Hydrogen bond-forming residues of wild SusD with cyclodextrin over the 200 ns simulation. Residues 172nd to 174th showed consistent interactions throughout, while residues 130th contributed up to 130 ns with minor fluctuation. Additional transient interactions were observed with residues 136th, 176th, and 178th. (B) Mutant SusD residues forming hydrogen bonds with cyclodextrin. Residues 448th and 453rd maintained notable interactions throughout, while residue 174th interacted stably up to 20 ns. Residues 444th and 450th to 452nd exhibited intermittent bonding.
